# Anticholinesterase, Antioxidant, Antiaflatoxigenic Activities of Ten Edible Wild Plants from Ordu Area, Turkey

**Published:** 2018

**Authors:** Belma Zengin Kurt, Işıl Gazioğlu, Ece Sevgi, Fatih Sönmez

**Affiliations:** a *Department of Pharmaceutical Chemistry, Faculty of Pharmacy, Bezmialem Vakif University, Istanbul, Turkey. *; b *Department of Analytical Chemistry, Faculty of Pharmacy, Bezmialem Vakif University, Istanbul, Turkey.*; c *Department of Pharmaceutical Botany, Faculty of Pharmacy, Bezmialem Vakif University, Istanbul, Turkey. *; d *Department of Chemistry, Faculty of Arts and Science, Sakarya University, Sakarya, Turkey.*

**Keywords:** Anticholinesterase activity, Antioxidant activity, Antiaflatoxigenic activity, Rumex acetosella, Capsella bursa-pastoris, Plantago lanceolata

## Abstract

Turkey has highly rich floras of medicinal and aromatic plants because of having various climate conditions in different regions. One of these regions is Middle Black Sea Region, especially Ordu Province. Extracts of 10 edible plants (*Arum maculatum *L., *Hypericum orientale *L., *Ornithogalum sigmoideum *Freyn et Sint., *Silene vulgaris *Garcke var. *macrocarpa*, *Plantago lanceolata *L., *Achillea millefolium *L. subsp. *pannonica*, *Rumex crispus *L., *Rumex acetosella *L., *Capsella bursa-pastoris *L., *Coronopus squamatus *Asch.), grown in Ordu, Turkey, were prepared with different solvents (hexane, ethanol and water, separately) and their anticholinestrase and antiaflatoxigenic activities were evaluated. Additionally, the cupric reducing antioxidant capacities (CUPRAC) and ABTS cation radical scavenging abilities of the extracts were assayed. The ethanol extract of *R. acetosella *exhibited the highest antioxidant activity (A_0.5_ value of 25.31 µg/mL, for CUPRAC activity; IC_50_ value of 23.73 µg/mL, for ABTS activity). The hexane extract of *C. bursa-pastoris *showed the strongest inhibition against AChE enzyme with IC_50_ value of 7.24 µg/mL, and the hexane extract of *A. millefolium *subsp. *pannonica *had the highest BChE activity with IC_50_ value of 6.40 µg/mL. The ethanol extract of *P. lanceolata *exhibited the strongest inhibition against aflatoxin with 88% inhibition.

## Introduction

Antioxidants play an important role as a health protecting effect for human wellness. These compounds reduce the risk for diseases including cancer and heart disease. It is known that the antioxidants (vitamin C, vitamin E, carotenes, phenolic acids *etc.*) in natural-sourced foods have a reducing effect on disease risk such as cancer and heart diseases *etc. *(1). Most of the antioxidant compounds in a typical diet are derived from natural sources and belong to various classes of compounds with a wide variety of physical and chemical properties (2). The free radical scavenging activity of antioxidants in foods has been reported by Miller *et al. *(3). Various antioxidant activity assays have been used to monitor and compare the antioxidant activity of foods using analytical techniques. These analytical techniques measure the radical scavenging activity of antioxidants against cation radicals like the [2,2’-azinobis(3- ethylbenzothiazoline-6-sulfonic acid)] (ABTS) or base on copper (II) reduction (CUPRAC) method (4).

Alzheimer′s disease (AD) is a physical disease that affects the brain. During the run of the disease, proteins established in the brain to form structures are called ′plaques′ and ′tangles′. This leads to the loss of connections between nerve cells, and eventually to the death of nerve cells and loss of brain tissue (5). Acetylcholinesterase (AChE; EC 3.1.1.7) is a hydrolase, the main role of which is to terminate the impulse transmission at cholinergic synapses by rapid hydrolysis of the neurotransmitter ACh. AChE inhibitors (AChEI) can inhibit the hydrolysis of ACh, and then improve the level of ACh in the specific regions of the brain (5, 6). Another cholinesterase, butyrylcholinesterase (BuChE; EC 3.1.1.8), is primarily found in plasma, liver, and muscle tissues. BuChE is metabolic degradation of ACh and differs from AChE for tissue distribution and sensitivity to substrates and inhibitors (7).

Recent studies showed that there was a relation between antioxidant property and cholinesterase inhibitory activity (8). AD is probably associated with multifaceted etiologies and pathogenic phenomena, and all mechanisms seem to share oxidative stress as a unifying factor, which is thought to have a causative role in the pathogenesis of AD recently (9). For this reason, natural products are the most important source of antioxidants which can be beneficial for treatment of Alzheimer’s disease.

Various chemical structures of mycotoxins, are the secondary metabolites, and modes of their actions are produced by various fungal species. There are more than 300 known mycotoxins classified as hepatotoxins, nephrotoxins, neurotoxins, and immunotoxins by clinicians, and as teratogens, mutagens, carcinogens, and allergens by cell biologists (10). Common mycotoxins include aflatoxins, ochratoxin A, ergot alkaloids, fumonisins, patulin, trichothecenes, and zearalenone (11). Aflatoxins (AFs) have the most acute toxic effects in humans and carcinogenic effects in susceptible animals among all mycotoxins (12). AFs, which are difuranocoumarin derivatives, are the toxic metabolites generated by the genus *Aspergillus *that include *A. flavus, A. parasiticus, A. nomius, A. tamari, and A. bombycis*. The major AFs, B1 (AFB1), B2 (AFB2), G1 (AFG1), and G2 (AFG2), are mainly present in cereals, peanuts, corn, nuts, and cottonseeds, and also the order of their toxicity is AFB1 > AFG1 > AFB2 > AFG2 (13). The International Agency for Research on Cancer (IARC) classified AFB1 as a human carcinogen (Group 1), and AFB2, AFG1, and AFG2 as possibly carcinogenic to humans (Group 2B). AFs also have toxigenic, mutagenic, and teratogenic effects (14). Because of these hazardous properties of aflatoxins, scientists have continued to search the new natural and/or synthetic aflatoxin inhibitors.

Turkey has highly rich floras of medicinal and aromatic plants because of having various climate conditions in different regions (15, 16). One of these regions is Middle Black Sea Region, which has highly plant diversity and rich cultures, especially Ordu Province (17). Ordu is situated within the Euxianian section of the Euro-Siberian floristic region (18-22).

This region has a great deal of wild edible plants. It is known that they have many medicinal properties (Table 1) (23-25).

An ethnopharmacobotanical study has revealed that there is a strong relationship between the medicinal plants and food medicine (26). *Arum *spp., *Capsella bursa-pastoris*, *Ornithogalum sigmoideum, Plantago lanceolata, Rumex acetosella, Rumex crispus *and *Silene vulgaris *are known to be used as foodstuff in many different regions of Turkey (16, 20, 24 and 27-29).

The leaves of *Plantago *species are also used for the treatment of wound and maturation of abscess in Turkey. Moreover *P. lanceolata *shows a significant constipation, expectorant, and diuretic activitiy (30).

*Rumex *spp. is used as poultice for maturation of fruncle in Turkey. The roots of *Rumex acetosella *have diuretic, cholagogue, and antipyretic activity. In addition, *Rumex crispus *has tonic, blood cleansing, digestive, and laxative effects (30).

*S. vulgaris *is used for bladder diseases, urinary tract infection in folk medicinal and *A. millefolium *has various biological activities such as diuretic, appetizing, carminative, emmenagogic, and wound healing for hemorrhoids (30). The foodstuff and medicinal uses of these edible plants are recorded in the ethnobotanical studies in Turkey (Table 1).

In this report, ten edible plants *(Arum maculatum *L., *Ornithogalum sigmoideum *Freyn et Sint., *Rumex acetosella *L., *Capsella bursa-pastoris *L., *Hypericum orientale *L., *Achillea millefolium *L. subsp*. pannonica, Silene vulgaris *Garcke var. *macrocarpa, Coronopus squamatus *Asch., *Plantago lanceolata *L., *Rumex crispus *L.) (1-10) were selected by considering the uses of them in traditional medicines in Ordu Area, Turkey. The extracts of them were prepared with hexane, ethanol and water, individually, and their antioxidant, anticholinesterase, and antiaflatoxigenic activities were experimentally evaluated.

## Experimental

*Plant material*

Plant samples were collected from Ordu in 2014. The specimens were prepared according to established herbarium techniques and identified according to Flora of Turkey and the East Aegean Islands (18, 31- 35). The voucher specimens were deposited in the Herbarium of Istanbul University, Faculty of Forestry (ISTO), Istanbul, Turkey.

*Chemicals and instrumentation*

The electric eel acetylcholinesterase (AChE, Type-VI-S, EC 3.1.1.7, 425.84 U/mg), horse serum butyrylcholinesterase (BChE, EC 3.1.1.8, 11.4 U/mg), 2,2-Azinobis (3- ethylbenzothiazoline- 6- sulfonic acid) diammonium salt (ABTS) (≥97.5%), acetylthiocholine iodide (≥98%), and potassium persulphate (≥99.99%) were purchased from Sigma-Aldrich (Germany). 5,5-dithiobis-(2-nitro benzoic acid) (DTNB) (≥98%), copper (II) chloride dihydrate (CuCl2.2H2O) (≥99%), neocuproine (2,9-dimethyl-1,10- phenanthroline) (≥98%) were obtained from Sigma (Germany). Butyrylthiocholine iodide (≥99%) was purchased from Fluka (Germany). Ridascreen Fast Aflatoxin (Art No.: R5202) test kit (Art No.: R1211) was obtained from R-Biopharm (Darmstadt, Germany). A Hanna pH-meter (USA), a Bandelin Sonorex ultrasonic bath (Berlin, Germany), a vortex (LMS, Japan), and a BioTek Power Wave XS ELISA Reader (USA) were used for the activity assays. All solvents were to be analytical grade.

*Preparation of Extracts*

The fresh plant samples were dried at the room temperature. Then, the dried plant materials (50 g) were chopped up and separated to three parts. They were macerated three times with 250 mL of hexane, ethanol, and water for 12 h, individually. After filtration of each extract, the solvents were evaporated to dryness *in-vacuo*, and the crude extracts were obtained, separately. The yield of the extracts varied from 0.54 to 11.73% (w/w) (Table 2).

*Determination of Anticholinesterase Activity of Plant Extracts*

AChE and BChE inhibitory activities were measured according to literature (36). Acetylthiocholine iodide and butyrylthiocholine iodide were used as substrates of the reaction and DTNB were used for the measurement of the anticholinesterase activity. All extracts were dissolved in ethanol to prepare their stock solution at 4000 µg/mL concentration. Aliquots of 130 mL of 100 mM sodium phosphate buffer (pH 8.0), x µL (four different concentration) of sample solution, and 20 µL AChE (or BChE) solution were mixed and incubated for 15 min at 25 ºC, and 10 µL of DTNB was added. The reaction was started by the addition of 20 µL acetylthiocholine iodide (or butyrylthiocholine iodide). Galantamine was used as a standard compound and ethanol was used as a control. The absorbance was measured at 412 nm by a 96-well microplate reader after 30 min.

*Determination of Antioxidant Activity of Plant Extracts*

BHA and ethanol were used as a standard and control, respectively, in both antioxidant assays.

*CUPRAC assay*

Antioxidant activity of the extracts was determined by CUPRAC assay according to the method described by Apak *et al. *(37). All plant extracts were dissolved in ethanol at 1000 µg/mL concentration. Aliquots of 61 µL of 1.0 × 10^−2^ M copper (II) chloride, 61 µL of ammonium acetate buffer (1 M, pH 7.0), and 61 µL of 7.5 × 10^−3^ M neocuproine solution were mixed, x µL sample solution (2.5, 6.25, 12.5, and 25 µL) and (67 − x) µL distilled water were added to make the final volume 250 µL. After 1 h, the absorbance was measured at 450 nm.

**Table 1:** Some Edible plants in Ordu

**Specimen** **Number** **(ISTO)**	**Botanical name**	**Family**	**Local name**	**Locality**	**Recorded literature traditional uses**
36723	*Achillea millefolium *L. subsp. *pannonica* (Scheele) Hayek (*Achillea seidlii *J.Presl and C.Presl.)	Asteraceae	Bereketotu	Çambaşı plateau, Ordu, 1800 m	Prostatitis, Headeache (47)
36716	*Arum maculatum *L.	Araceae	Nivik	Doğanlı village, Perşembe- Ordu, 10 m	Foodstuff (20), Foodstuff for hemorrhoid (24)
36724	*Capsella bursa-pastoris *(L.) Medik.	Brassicaceae	Gazayağı	Çambaşı plateau, Ordu, 1800 m	Diabetes (47), Foodstuff (20, 27, 28 and 16), Kidney stones, analgesic for pain in the kidney (24), Constipation, diuretic (48, 49) Foodstuff for intestine (50) Impotence (51)
36721	*Coronopus squamatus *(Forssk.) Asch. (*Lepidium coronopus *(L.) Al-Shehbaz)	Brassicaceae	Kedidili	Çambaşı plateau, Ordu, 1800 m	-
36725	*Hypericum orientale *L.	Guttiferae	Yayla kekiği	Çambaşı plateau, Ordu, 1800 m	Sedative (52)
36714	*Ornithogalum sigmoideum *Freyn et Sint.	Liliaceae	Sakarca	Altınyurt village, Ordu, 480 m	Foodstuff (20, 24)
36722	*Plantago lanceolata *L.	Plantaginaceae	Kurtbaşı	Çambaşı plateau, Ordu, 1800 m	Burns, antiasthmatic, cut, contusion, eczema (47), Wound (47, 51), Gastrointestinal diseases (49), Astringent (49, 51 and 52), Ulcer (51), Maturation of abscess, against pimple (23)
36720	*Rumex acetosella *L.	Polygonaceae	Kuzukulağı	Çambaşı plateau, Ordu, 1800 m	Foodstuff (27, 28 and 16) Diabetes, hypertension (51)
36717	*Rumex crispus *L.	Polygonaceae	Katırtırnağı	Çambaşı plateau, Ordu, 1800 m	Foodstuff (27, 28), Scabies (53)
36715	*Silene vulgaris *(Moench) Garcke var. *macrocarpa *(Turrill) Coode et Cullen	Caryophyllaceae	Gıcırık	Çambaşı plateau, Ordu, 1800 m	Foodstuff (27, 28 and 16)

**Table 2:** Yields of the extracts (w/w%).

**Sample code**	**Plant name**	**Extract**		
		**Hexane**	**Ethanol**	**Water**
1	*Arum maculatum*	0.08	0.29	3.86
2	*Ornithogalum sigmoideum*	0.08	0.54	0.98
3	*Rumex acetosella*	1.76	2.99	5.46
4	*Capsella bursa-pastoris*	3.00	5.26	7.45
5	*Hypericum orientale*	4.50	11.73	6.03
6	*Achillea millefolium*	1.96	3.80	8.15
7	*Silene vulgaris*	1.92	4.45	10.46
8	*Coronopus squamatus*	2.21	3.21	1.98
9	*Plantago lanceolata*	1.25	0.91	2.48
10	*Rumex crispus*	1.00	1.60	2.14

**Table 3:** *In-vitro *inhibition IC50 and A0.5 values (μg/mL) for AChE, BChE, antioxidant and antiaflatoxigenic activities of the extracts

**Plant**	**Extract**	**ABTS** ^·+^ ** assay** **IC**_50_ ** (µg/mL)** b	**CUPRAC assay A**_0.50_ ** (µg/mL)** c	**AChE assay** **IC**_50_ ** (µg/mL)** b	**BChE assay** **IC**_50_ ** (µg/mL)** b	**Anti- aflatoxigenic activity (inhibition %)**
	hexane	153.99 ± 0.15	>200	128.01 ± 0.53	23.73 ± 0.09	19.73
1	ethanol	143.53 ± 0.23	190.03 ± 0.04	149.65 ± 0.42	>200	28.94
	water	106.01 ± 0.42	>200	>200	142.78 ± 0.21	21.05
	hexane	>200	>200	>200	40.85 ± 0.68	40.78
2	ethanol	>200	>200	154.01 ± 0.93	>200	14.47
	water	>200	>200	>200	>200	21.05
	hexane	>200	201.45 ± 0.05	120.08 ± 0.75	51.50 ± 0.34	47.36
3	ethanol	23.73 ± 0.65	25.31 ± 0.02	>200	52.55 ± 0.35	42.10
	water	47.45 ± 0.27	68.47 ± 0.04	82.74 ± 0.56	72.23 ± 0.94	23.68
	hexane	>200	157.88 ± 0.07	7.24 ± 0.08	21.15 ± 0.15	73.68
4	ethanol	174.98 ± 0.89	>200	>200	89.45 ± 0.63	32.89
	water	66.14 ± 0.74	168.13 ± 0.07	>200	>200	32.89
	hexane	114.49 ± 0.12	124.80 ± 0.06	>200	>200	18.42
5	ethanol	54.92 ± 0.32	67.65 ± 0.06	165.20 ± 0.62	50.57 ± 0.32	7.89
	water	42.39 ± 0.54	58.06 ± 0.02	>200	>200	5.26
	hexane	>200	>200	27.24 ± 0.46	6.40 ± 0.08	63.15
6	ethanol	149.36 ± 0.98	171.76 ± 0.02	>200	49.57 ± 0.76	39.47
	water	83.31 ± 0.35	117.63 ± 0.05	119.72 ± 0.38	>200	40.78
	hexane	>200	>200	>200	>200	47.36
7	ethanol	199.02 ± 0.78	>200	95.13 ± 0.13	>200	28.94
	water	91.59 ± 0.41	76.72 ± 0.07	NC^a^	>200	17.10
	hexane	>200	198.67 ± 0.04	NCa	107.79 ± 0.48	34.21
8	ethanol	91.31 ± 0.25	51.77 ± 0.08	50.12 ± 0.26	NC^a^	52.63
	water	>200	115.04 ± 0.04	NC^a^	117.26 ± 0.86	21.05
	hexane	>200	>200	NC^a^	>200	30.26
9	ethanol	39.28 ± 0.39	40.65 ± 0.08	>200	195.86 ± 0.99	88.15
	water	>200	57.02 ± 0.01	>200	192.46 ± 0.21	43.42
	hexane	62.08 ± 0.56	>200	36.98 ± 0.89	93.42 ± 1.96	52.63
10	ethanol	42.85 ± 0.47	14.92 ± 0.07	57.82 ± 0.35	>200	57.89
	water	40.22 ± 0.78	23.10 ± 0.06	82.44 ± 0.47	72.14 ± 0.64	1.3
	dBHA	1.18 ± 0.03	1.45 ± 0.02	-	-	-
	eGalantamine	-	-	0.5 ± 0.01	17.4 ± 0.03	-

a NC.: not calculated.

b IC_50_ values represent the means ± SEM of three parallel measurements (*p *< 0.05).

c A_0.50_ values represent the means ± SEM of three parallel measurements (*p *< 0.05).

d Reference compound for antioxidant assays.

e Reference compound for anticholinesterase assays.

*ABTS* ^+.^ *Cation Radical Decolorization Assay*

ABTS^+.^ scavenging activities of the extracts were determined according to the literature (38). The solution of ABTS^+.^ radical was generated by dissolving 19.2 mg of 2,2-azino-bis(3-ethylbenzothiazoline- 6-sulphonic acid) (7 mM ABTS) and 3.3 mg K_2_S_2_O_3_ in distilled water (5 mL). The solution was kept in dark for 24 h at room temperature, and the absorbance of the solution was fixed to 0.70 at 734 nm by dilution. The solutions of the samples were prepared in n-propanol at a concentration of 1000 µg/mL. The absorbance was measured in room temperature at 734 nm, after 6 min from ABTS^+^.addition. The decrease in the absorption was used to calculate the activities.

*Antiaflatoxigenic activity assay*

Fifty microlitres of the standard and extracts were transferred into separate wells. Firstly, aflatoxin standard (45 µg/mL) was added to all extract wells. Then, the solutions of enzyme conjugate and the antiaflatoxin antibody (50 μL of each one) were added to each well, respectively. After shaking and incubating the plate at room temperature for 10 min, the liquid was removed from the wells. The wells were filled with PBS and all the remaining liquid was removed. The washing step was repeated two more times. Then, 100 μL of the substrate/chromogen was added to each well. The plate was shaked and incubated at room temperature in the dark for 5 min. Finally, 100 μL of the H_2_SO_4_ (%10) was added to each well and shaked. The absorbance was measured at 450 nm and processed with RIDA®SOFT Win (Art. No. Z9999) software, dedicated to the Ridascreen® procedure. The inhibition powers were calculated against the absorption of aflatoxin standard (39).

*Analytical quality assurance*

The results are given as mean ± standard deviation of the mean. Statistical analyses of the data were performed with Student *t*-test. *p*-values lower than 0.05 were considered as statistically significant.

## Results and Discussion

The selected plants belong to different families, such as Araceae, Liliaceae, Polygonaceae, Brassicaceae, Plantaginaceae, *etc. *It is known that different solvents can extract different molecules, depending on their polarities and solubilities, from plants. So the plant materials were separately extracted with hexane, ethanol, and water by maseration and biological activities of these extracts were analyzed and compared with different polarities. The yields of the extracts are given in Table 2.

Antioxidant activities of the extracts were examined using spectrophotometric assays with the ABTS cation radical scavenging activity and CUPRAC (37). Most of the extracts showed acceptable antioxidant activities (Table 3). Among the extracts, the ethanol extract of *R. acetosella *(3) had the highest antioxidant potential for ABTS and CUPRAC with IC_50_ value of 23.73 µg/mL and 25.31 µg/mL, respectively. Isbilir and Sagiroglu have reported that *R. acetosella *includes rutin, flavone glycosides, vitamins, carotenoids, minerals, and phenolics, so it has various biological acitivites such as antioxidant, anticancer, antidiabet, and anti-inflammatory effects. Additionally, the methanol extract of *R. acetosella *showed the highest antioxidant activity with IC_50_ value of 0.03 µg/mL in DPPH assay in this report (40). Also, Alpinar *et al. *tested antioxidant capacity of *R. acetosella *using CUPRAC method (0.12 ± 0.03 mmol g^-1^) and they found similar results with our study (41). It is known that *Rumex *genus is rich in anthracene derivatives. Wegiera *et al. *identified that *Rumex *species contain chrysophanol, physcion, emodin, aloe-emodin, barbaloin, sennoside A, and sennoside B, as anthracene derivatives oftheir studies showed that the antioxidant potentials of anthrecene derivatives were determined with various methods such as DPPH radical scavenging ability, reducing power, and superoxide scavenging activity (42).

On the other hand, the ethanol extract of *P. lanceolata *(9) exhibited low to moderate antioxidant activity for ABTS and CUPRAC with IC_50_ value of 39.28 µg/mL and 40.65 µg/mL, respectively. Ferrezzano *et al. *have reported that methanol : water extract of *P. lanceolata *includes phenolic acids, flavonoids, coumarins, lignans, lipids, and cinnamic acids (43). The antioxidant effect of this species could be related to contained substances and mostly like the results of the synergic activity of the main compounds. The antioxidant effects of *O. sigmodeum *(2), *S. vulgaris *(7), *A. millefolium *subsp. *pannonica *(6) and *C. squamatus *(8) species have not been investigated up to now. As seen in Table 3, the results of antioxidant assays showed that the all extracts were showed moderately antioxidant activity.

The inhibitory activities of the extracts of selected species on AChE and BChE were determined by the Ellman’s method using galantamine as the reference compound (36). The IC_50_ values for AChE and BChE inhibitions are summarized in Table 3. IC_50_ values against AChE are between 7.24 µg/mL and 165.20 µg/mL. IC_50_ values against BChE are between 6.40 µg/mL and 142.78 µg/mL.

The anticholinesterase activity (against acetyl- and butryl-cholinesterase enzymes) of extracts was compared with that of galantamine (IC_50_ = 0.5 ± 0.01), used as a standard drug against Alzheimer’s disease. Among them, the hexane extract of *Capsella bursa-pastoris *(4) has the highest inhibitory activity against AChE (IC_50_ = 7.24 ± 0.08).

*C. bursa-pastoris *has various biological activities such as antibleeding, anticancer, and

antithrombin, *etc. *because of containing minerals, vitamins, ascorbic acid, proteins, linoleic acid, fatty acids (44). Grosso *et al. *investigated the metabolic profile of *C. bursa-pastori*s with methanol extract and quercetin-6-C-glucoside, kaempferol-3-O-glucoside, fatty acids, organic acids, aminoacids, and sterols were determined. According to the presented study by Grosso *et al.*, the methanol extract of *C. bursa-* *pastori*s had moderate inhibitory activity against AChE (19% inhibition at a concentration of 0.1 mg/mL) (44).

The hexane extract of *A. millefolium *subsp*. pannonica *(6) had the highest BChE inhibitory activity with IC_50_ value of 6.40 µg/mL, in our study. Karaalp *et al. *have previously reported that *Achillea *species include flavonoids and sesquiterpen lactones. It is known that flavonoids have anticholinesterase properties and several biological activities (45). Also, sesquiterpene lactones have various biological activities such as antiinflammatuar, antipyretic, antimalarial, antimicrobial, antiviral, antiphytoviral, antiulserogenic, smooth muscle contractility, immunologic, neurotoxic, and cytotoxic (46). It can be said that due to synergic interactions between these compounds and *A. millefolium*, this species might be an effective cholinesterase inhibitor.

Sixteen percent of the all plant extract samples have minimal or no cholinesterase inhibitory activity. This result showed that most of the active molecules against AChE and BChE were in hexane (nonpolar solvent) extract. This may be most likely due to anticholinesterase activity of nonpolar compounds found in high amounts within these extracts. The studying of nonpolar extracts for anticholinesterase activity are worth further phytochemical evaluation for identifying their active components.

Antiaflatoxigenic activities of the extracts of all selected plants were tested *in-vitro *according to R-Biopharm Elisa Fast aflatoxin method (39). The results of antiaflatoxigenic assay showed that the ethanol extract of *P. lanceolata *and *A. millefolium *subsp*. pannonica *exhibited good potential to reduce the amount of aflatoxin.

## Conclusion

The selected ten edible plants *(A. maculatum *L., *H. orientale *L., *O. sigmoideum *Freyn et Sint., *S. vulgaris *Garcke var. *macrocarpa, P. lanceolata *L., *A. millefolium *L. subsp*. pannonica*, *R. crispus *L., *R. acetosella *L., *C. bursa-pastoris *L. *C. squamatus *Asch.), grown in Black Sea Region of Turkey, were extracted with hexane, ethanol and water, individually, and their antioxidant, anticholinesterase, and antiaflatoxigenic activities were investigated. The anticholinesterase and antiaflatoxigenic activities of all selected species were examined for the first time by the presented study. The ethanol extract of *R. acetosella *exhibited the highest antioxidant activity (A0.5 value of 25.31 µg/mL, for CUPRAC activity; IC_50_ value of 23.73 µg/mL, for ABTS activity). The hexane extract of *C. bursa-pastoris *showed the strongest inhibition against AChE enzyme with IC_50_ value of 7.24 µg/mL, and the hexane extract of *A. millefolium subsp. pannonica *had the highest BChE activity with IC_50_ value of 6.40 µg/mL. The ethanol extract of *P. lanceolata *exhibited the strongest inhibition against aflatoxin with 88% inhibition. The results indicated that most of the extracts of the selected plants inhibited both ChEs and aflatoxins, and also had antioxidant activity. Polar extracts such as ethanol and water, possessed strong activity in all assays, because of probably containing phenolic compounds. Also, the high anticholinesterase, significant antioxidant, and aflatoxin inhibitory activities of these some plants bring lots of advantages for its application in manifucturing food supplements.

The obtained results confirm the antioxidant, anticholinesterase, and antiaflatoxigenic activity of edible wild plants from Ordu and thus these plants can be used as source of natural food supplements.
